# Evaluation of the Accuracy, Credibility, and Readability of Statin-Related Websites: Cross-Sectional Study

**DOI:** 10.2196/42849

**Published:** 2024-03-14

**Authors:** Eunice Ling, Domenico de Pieri, Evenne Loh, Karen M Scott, Stephen C H Li, Heather J Medbury

**Affiliations:** 1 Sydney Medical School, Faculty of Medicine and Health The University of Sydney Sydney Australia; 2 Institute of Clinical Pathology and Medical Research Westmead Hospital Westmead Australia; 3 Vascular Biology Research Centre Surgery Westmead Hospital Westmead Australia

**Keywords:** statins, consumer health information, readability, credibility, accuracy, digital health, health information seeking, cardiovascular, mortality, management, pharmacotherapy, risk, medication

## Abstract

**Background:**

Cardiovascular disease (CVD) represents the greatest burden of mortality worldwide, and statins are the most commonly prescribed drug in its management. A wealth of information pertaining to statins and their side effects is on the internet; however, to date, no assessment of the accuracy, credibility, and readability of this information has been undertaken.

**Objective:**

This study aimed to evaluate the quality (accuracy, credibility, and readability) of websites likely to be visited by the general public undertaking a Google search of the side effects and use of statin medications.

**Methods:**

Following a Google web search, we reviewed the top 20 consumer-focused websites with statin information. Website accuracy, credibility, and readability were assessed based on website category (commercial, not-for-profit, and media), website rank, and the presence or absence of the Health on the Net Code of Conduct (HONcode) seal. Accuracy and credibility were assessed following the development of checklists (with 20 and 13 items, respectively). Readability was assessed using the Simple Measure of Gobbledegook scores.

**Results:**

Overall, the accuracy score was low (mean 14.35 out of 20). While side effects were comprehensively covered by 18 websites, there was little information about statin use in primary and secondary prevention. None of the websites met all criteria on the credibility checklist (mean 7.8 out of 13). The median Simple Measure of Gobbledegook score was 9.65 (IQR 8.825-10.85), with none of the websites meeting the recommended reading grade of 6, even the media websites. A website bearing the HONcode seal did not mean that the website was more comprehensive or readable.

**Conclusions:**

The quality of statin-related websites tended to be poor. Although the information contained was accurate, it was not comprehensive and was presented at a reading level that was too difficult for an average reader to fully comprehend. As such, consumers risk being uninformed about this pharmacotherapy.

## Introduction

### Background

Cardiovascular diseases (CVDs) are the primary cause of death globally, with an estimated 17.9 million people dying of CVDs in 2021. This represents 31% of all global deaths. Of these deaths, 85% are due to heart attack and stroke, whose most common etiology is atherosclerosis [1]—the development of fatty plaque within artery walls. A key pharmacological treatment for atherosclerosis is statin therapy. It has a role in the primary and secondary prevention of vascular events, with a lowering of low-density lipoprotein cholesterol leading by 2 mmol/L, typically giving a 10% absolute benefit (the reduction in the probability of an event’s occurrence within a population receiving treatment) for those diagnosed with vascular disease and a 5% absolute benefit for those with risk factors yet without having experienced a vascular event [2]. This creates issues when we consider that patients may be biochemically abnormal (with hypercholesterolemia) but asymptomatic. Such patients may doubt the use of the prescribed statin therapy as they determine the cost-benefit balance between tangible adverse effects and theoretical benefits. This may prompt the consultation of alternative sources of knowledge to aid decision-making. In this era of shared decision-making, where patients participate in the medical decisions that affect their health [3], it is essential that the information they access is high quality and easily understood.

### Use of the Internet for Health Information Seeking

In this milieu, the internet has risen as a key source of health-related information, with 79% of adults seeking web-based health information in America and 79% to 86% in China, the Philippines, Hong Kong, Indonesia, and Vietnam [4,5]. Comparatively, seeking web-based health information is as popular as playing games or downloading music from the web [6]. Notably, the COVID-19 pandemic has presented unprecedented challenges, catapulting society further into a future dependence on telehealth and internet-assisted health care [7,8]. As such, traditional in-clinic and leaflet modes of health information delivery are being supplemented, and in some cases supplanted, by internet searches. With this dramatic change in the terrain upon which patients and their families are attaining information, it is crucial to determine the quality of web-based health information put forth to them.

Health literacy is defined as “the degree to which individuals have the capacity to obtain, process, and understand basic health information and services needed to make appropriate health decisions” [9]. It requires a complex group of skills such as reading, listening, analyzing, and decision-making, as well as the ability to apply the aforementioned skills to health situations [10]. Those with poor health literacy are vulnerable to undertaking unnecessary tests and treatments or, conversely, refusing beneficial tests and treatments. In part, they may be misled into assessing the quality of web-based health information based on its search result ranking, image quality, celebrity endorsement, and website authorship rather than relying on the criteria of established quality guidelines [11,12].

Health literacy–related knowledge and skills are particularly deficient among vulnerable populations, who are also more likely to experience CVD [12,13]. Unfortunately, those with poor health literacy are susceptible to the influence of mass media and emotionally persuasive texts. This may explain the response to the television program “Catalyst” in Australia [14], whose criticism of statins resulted in 11% of patients (in a survey by the Australian National Heart Foundation of 1094 patients) who watched the program ceasing to take their cholesterol-lowering medication and significant and sustained changes in statin usage, with 2.6% fewer statins (equivalent to 14,005 dispensing) each week [15].

### Internet Standards

There is considerable heterogeneity in the quality of web-based health information [16]. The quality of information can be examined in 3 domains: accuracy, credibility, and readability [17]. Each is defined as follows: accuracy is the intent to be evidence based and safe by adequately offering a complete, unbiased picture and its relevance [17]; credibility is the attribution of source and authorship and the disclosure of conflicts of interest for the presented information [18]; and readability is the ease of understanding due to the style of writing, describing the reading comprehension level a layperson requires to understand a text [19,20]. Complex wording reduces engagement with, and application of, content [21]. This leaves patients vulnerable to becoming ill-informed and at risk of adverse health outcomes [22]. The quality of web-based health information is, therefore, a pressing issue, which the Health on the Net Foundation aims to address by providing a Health on the Net Code of Conduct (HONcode) seal, an internet-based certification of medical and health websites that adheres to a set of publishing principles regarding the source and purpose of medical information. However, few consumers are aware of Health on the Net or the HONcode.

While there have been assessments of the quality of websites on many key areas of health care, such as diabetes [23], obesity [24], and hypertension [25], and on surgical interventions [26,27], there is a dearth of assessment about the quality of web-based information pertaining to medications. Such assessments are necessary to inform clinicians of the quality of content likely to be accessed by their patients, who are particularly interested in the likelihood and nature of adverse side effects. We aimed to assess the quality of consumer health information on websites about statins through consideration of accuracy, credibility, and readability.

## Methods

### Study Design

#### Overview

Through this cross-sectional study, we analyzed the accuracy, credibility, and readability of websites that were most commonly presented to patients searching for the keywords “statin” and “statin side effects.” We determined each website’s search engine ranking, category (commercial, not-for-profit, or media), and the presence or absence of the HONcode seal. Furthermore, we determined the relationship between the accuracy, credibility, and readability of the websites found.

In selecting websites on statins to analyze, we aimed to emulate a typical consumer’s search for web-based health information. A web search was conducted using Google, in keeping with evidence that it accounts for more than half of all web traffic [28-30] and is an increasingly preferred search engine by the general public [31]: 91% of American adults using the internet use a search engine, and of those, 83% use Google more often than other search engines [4,32]. To conduct the search, location filters, user information, search history, cached data, and cookies were disabled, and sponsored results were excluded to avoid inadvertent search bias. The search terms used were “statin” and “statin side effects,” following the advice of our lipidologist coauthor (SL) that the generic term “statin,” rather than specific medication names, was commonly used in discussion with patients in clinical practice and that “statin side effects” were a key concern of patients.

The first 20 ranked websites in the Google search results page were analyzed (after removing any duplication from search results of the 2 search terms). We did not identify further websites in the search results, given that, in general, websites returned on the first Google search results page generate 92% of all traffic from an average search [16,33]. This drops by 95% for the second page and by 78% and 58% for subsequent pages [31]. Thus, we did not aim to identify all websites on statins but rather to emulate an authentic consumer search.

#### Website Search Rank

The effect of the association between search result ranking and accuracy, credibility, and readability was considered. Given that engagement is highest with the first 5 websites in search results, garnering 67% of all clicks from a search results page [31,34], the websites were divided into 4 sets of 5 websites, each according to their ranking as per Google search result. Thus, websites ranked 1-5 were called quartile 1, websites 6-10 were called quartile 2, and so forth.

#### Website Categorization

In the interest of determining whether the nature of the authorship of the websites had a bearing on their accuracy, credibility, and readability, each of the 20 chosen websites was categorized into 3 types: commercial (defined as a website that generates revenue or cash and is not affiliated with the government), not-for-profit (a website that garners support for a cause rather than revenue, including government and charities), and media (a website that reports new findings or stories, with the primary purpose of the website being news reporting).

#### Presence of HONcode Seal

We assessed whether the quality of the websites was associated with the presence or absence of the HONcode seal.

### Measures

#### Overview

In assessing the accuracy, credibility, and readability of the websites, we considered existing tools and developed study-specific tools where necessary.

#### Accuracy

For accuracy, we developed a statin-specific tool that took into account medical guidelines. The checklist (Table 1) was designed after referring to other studies purporting to assess the quality of web-based health information [17]. A key difference here was that we were looking at a specific treatment. Three features were considered: (1) the intent to be evidence based; (2) safety, in that a website should adequately offer users a complete, unbiased picture of statin treatment; and (3) relevance, in that it is reasonable to expect a website to address the criteria in the checklist [17]. The checklist was intrinsically linked to a website’s comprehensiveness, consistent with other studies that have evaluated completeness as an integral part of accuracy [16].

To meet these features, guidelines from the American College of Cardiology and the Australian Heart Foundation [37] were synthesized into short statements, which formed the accuracy criteria. These statements formed a checklist that each website was required to address to be considered “accurate.” The development of the statements was further informed and determined by a review of the treatment of cholesterol in light of its evidence base [38], as well as criteria from the treatment section of the validated DISCERN tool [39]. This section (items 9-16 of the DISCERN tool) addresses issues of risk, benefit, and how the website guides decision-making surrounding treatment options [39]. Combining these sources ensured that a higher score would be awarded to websites providing the most evidence-based information. In total, 20 equally weighted criteria were devised, and a score of 20 was arbitrarily defined as a minimum acceptable standard.

Each accuracy item was scored as: “present and complete” (2), “present but incomplete” (1), “absent” (0), or “inaccurate” (*). A maximum score of 40 could be awarded to each website assessed. “Incompleteness” was defined as a nonexact or indirect mention of a topic outlined in a criterion rather than an explicit statement. Two reviewers (DdP and E loh) completed the assessment.

Any hyperlinks that navigated to information within a website were followed and the data were included in the final assessment; links leading to external websites were not followed. Embedded videos were analyzed. Once each reviewer concluded their analysis, the results were compared. Discrepancies were resolved through discussion until reaching a consensus.

**Table 1:** Website accuracy checklist.

Accuracy criteria	Descriptor
1	Mentions that cholesterol is a modifiable risk factor for cardiovascular disease [35]
2	Mentions that consultation with a doctor is essential before and while taking statins and when ceasing them [36]
3	Lists conditions for which statins are used [36]
4	Defines the target population for statin therapy [37]
5	Mentions the importance of adherence to statin therapy [36]
6	Addresses the subtleties of primary prevention [38]
7	Mentions that statins are about reducing complications of high cholesterol rather than achieving a specific (low-density lipoprotein) cholesterol [38]
8	Describes or at least lists the benefits of statin therapy [39]
9	Describes or at least lists the side effects or risks of statin therapy [39]
10	Describes how treatment affects the overall quality of life [39]
11	Mentions low to moderate dose statin therapy is recommended in primary prevention [37]
12	Specifically addresses rhabdomyolysis [36]
13	Describes the approximate financial burden to the patient [36]
14	Describes the duration of treatment before an effect is measurable [39]
15	Describes how statins work or at least what they do [39]
16	Describes what may happen without treatment [39]
17	Explores the possibility of using alternative therapies to statins [39]
18	Mentions that statins must not be used during pregnancy [37]
19	Describes drug interactions or at least lists them [39]
20	Mentions that statins do not replace a healthy lifestyle [36]

#### Credibility

In developing the criteria to be included in the assessment of credibility (Table 2), DISCERN was chosen as a reference, as well as other studies that used DISCERN or another available tool for website assessment. However, as the 5-point Likert scale used in DISCERN can be subject to response style bias [40], a present (1) or absent (0) scale was adopted as it has been shown to improve the objectivity of data collection [41-43].

**Table 2:** Website credibility checklist.

Item	Criteria	Score criteria
1	Referencing or citations obtained from peer-reviewed journals	1 point if the articles for which the references are obtained are published in peer-reviewed journals [16,44]
2	Website updated within last 24 months	The latest update should be within the past 24 months [45]
3	Avoids anecdotal evidence for making claims	Does not use anecdotal evidence as a basis for claims; quoting a case study without using claims is acceptable [46]
4	Mailing address present	Physical contact address of the website clearly stated [45]
5	Contact information available	Contact information including name, position, telephone number, address, and email [47]
6	Sponsorship stated	Any sponsorship should be clearly stated
7	Organizational privacy policy stated	Organization privacy policy should be clearly stated [47]
8	Declaration of the author’s qualification	Author’s qualification should be health care related [16,44]
9	Paid access tab present	If paid access is available, the difference in the information obtained from paid vs unpaid access should be clearly stated [48]
10	Disclosure of funding or conflicts of interest	Conflicts of interest and funding disclosure should be clearly stated [44]
11	The presence of an HONcode seal or third-party certification	Presence of a HONcode^a^ seal or any other third-party certification [16]
12	Advertisement neutral	Advertisements should steer clear from the website information (eg, no pop-ups related to the website content) [16]
13	Disclaimer regarding web-based health information	A disclaimer should be clearly stated that web-based health information does not replace a practitioner’s advice [44]

^a^HONcode: Health on the Net Code of Conduct.

Each website was appraised according to this list. A score was allocated for each website’s front page, with internal links explored only if relevant. Data (credibility scores) were undertaken as independent assessments by 2 assessors (E Loh and DdP). The results were compared, and if discrepancies arose, discussions were held to clarify the score, with external input from advisors (HM, SL, and KS) obtained where appropriate.

#### Readability

For readability, various tools are available, including the Flesch Kincaid Reading Ease, Flesch Kincaid Grade Level, Simple Measure of Gobbledygook (SMOG), and Average Grade Level. We used SMOG as it is considered the gold standard for assessing the readability of health care material and has a high correlation with the other scoring systems [24,49]. Importantly, the outcome measure is easy to understand as, for example, an SMOG readability grade of 6 represents a text comprehensible to all individuals with sixth-grade reading skills and above [50-53]. This grade level was set as the basis of readability, given that the available literature sets this as the standard for “superior” readability. To use this tool, texts from the 20 selected websites were copied and saved as separate Microsoft Word (Microsoft Corp) and plain text documents for analysis, deleting text unrelated to the health information topic (eg, author information or disclaimers) to prevent this from confounding the scoring. A single web-based readability calculator [54] was used to generate the scores.

### Data Analysis

The website category and ranking findings were compared by ANOVA, and differences between websites with and without the HONcode seal were analyzed with 2-tailed *t* tests. In addition, the relationship between credibility and readability with accuracy was assessed by Pearson correlation.

### Ethical Considerations

As the research was not conducted on human subjects, no ethics review was required.

## Results

### Selected Websites

The top 20 websites returned by the search are listed in Multimedia Appendix 1 [55-74]. Of the 20 websites chosen from the search, 45% (n=9) were categorized as commercial, 45% (n=9) not-for-profit, and 10% (n=2) media (Table 3). There was an even distribution of commercial and not-for-profit websites across the 4 quartiles, with both media websites found in the fourth quartile. Eight of the websites bore the HONcode seal.

**Table 3:** Top 20 statin websites’ category, HONcode^a^ presence or absence, accuracy, credibility, and readability.

Website rank	Category	HONcode Seal	Accuracy	Credibility	Readability (SMOG^b^ score)
1	Commercial	Yes	20	6	10.7
2	Not-for-profit	Yes	9	11	11.3
3	Not-for-profit	No	23	9	12.5
4	Commercial	Yes	16	6	13
5	Commercial	No	10	4	7.7
6	Not-for-profit	No	15	7	13
7	Commercial	No	14	10	9.6
8	Not-for-profit	No	13	5	10.8
9	Not-for-profit	No	16	11	10.2
10	Commercial	Yes	11	10	8.6
11	Commercial	Yes	16	9	9.4
12	Commercial	No	13	6	11
13	Not-for-profit	No	17	9	8.9
14	Not-for-profit	No	8	7	9
15	Not-for-profit	No	8	7	10.3
16	Media	No	8	4	7.8
17	Not-for-profit	Yes	16	11	6.7
18	Commercial	Yes	19	8	9.7
19	Commercial	Yes	21	7	9.2
20	Media	No	14	2	8.1

^a^HONcode: Health on the Net Code of Conduct.

^b^SMOG: Simple Measure of Gobbledegook.

### Accuracy

The mean website accuracy score was 14.35 (SD 4.43). In terms of accuracy, the 3 highest-scoring websites were Wikipedia (score of 23), Drugs.com (score of 21), and Medicine.net (score of 20). These were the only websites to achieve a score of 20 or above. No website contradicted any checklist criterion. The top 3 performing checklist criteria were related to side effects and statin mechanism of action (criteria 9, 12, and 15), with a score of “present and complete” for each of these criteria achieved by 18, 14, and 12 websites, respectively. Although side effects were covered to some degree in all websites, criteria about drug safety (criteria 18 and 19) were complete in only 8 and 7 websites, respectively. Other poorly performing criteria reflected the lack of detail about primary prevention (criteria 11 and 6), with a score of “absent” assigned to 19 and 17 of the websites, respectively.

### Credibility

None of the sampled websites met all credibility criteria for a perfect score of 13. The mean score overall was 7.45, with a range of 2-11. Importantly, 12 websites referenced peer-reviewed journal articles as a source of information, and 15 avoided anecdotal evidence for making claims. Media and some commercial websites reported personal opinions. While only 6 websites provided an organization’s contact details, the others provided an email address or feedback form for contact purposes. Sponsorship was explicitly stated in 12 websites, with reference to either government or private organizations. All websites declared their organization’s privacy policy, including websites with lower overall credibility scores. Only 8 websites declared author qualifications, which were primarily health related. None of the websites required paid access. Funding sources were fully disclosed in 11 websites, with the remaining 9 not reporting their source of funding or conflicts of interest. Twelve websites either had no advertisements or non–health care advertisements; the 8 websites that did not meet this criterion were commercial or media websites. Only 5 websites did not include a disclaimer that web-based health information does not replace a practitioner’s advice: all of these websites were commercial or media websites.

### Readability

Overall, for SMOG readability, the median was 9.65 (IQR 8.825-10.85) and the average was 9.875 (SD 1.75), that is, above the ninth-grade level. None of the websites met the recommended grade level of 6; even the media websites required an eighth-grade level of comprehension.

### Correlation Between Accuracy, Credibility, and Readability

**Figure 1:**
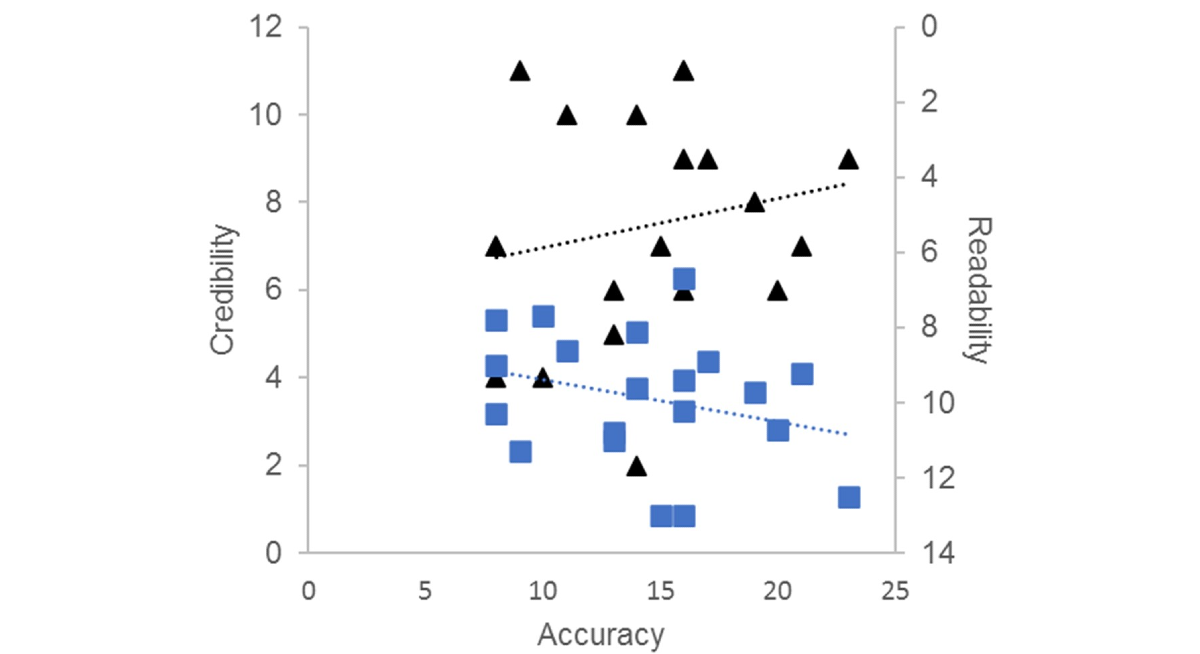
Relationship between website credibility (black triangles) and correlation readability (blue squares) with accuracy (*P*=.23).

No significant correlation was evident between the correlation between credibilty and accuracy (*P*=.23) (Figure 1).

### Website Search Rank

Websites that featured prominently in search results were not necessarily the most accurate, with no significant difference between the quartiles (*P*=.64). Indeed, of the 20 websites reviewed, the second highest scoring website for accuracy was ranked in the 19th position on search results. Similarly, there was no difference in credibility (*P*=.63) or readability (*P*=.06) between the quartiles.

### Website Categorization

Comparing commercial with not-for-profit websites, 2-tailed *t* tests revealed there was no significant difference in terms of accuracy (*P*=.275), credibility (*P*=.83), or readability (*P*=.452). As there were only 2 media websites, a comparison with them was not made. Notably, they had the lowest scores for credibility, but both scored among the most readable.

### Presence of HONcode Seal

Of the 20 websites, 8 were HONcode certified, with 6 of these categorized as commercial websites and 2 as not-for-profit. The mean accuracy scores for websites with and without the HONcode seal were 16 (SD 4.2) and 13.25 (SD 4.4), respectively, but this was not significantly different (*P*=.18). The presence or absence of the HONcode seal did not preclude a website from scoring at either end of the accuracy scale. Although the 8 websites with the HONcode seal scored higher in credibility (mean 8.5, SD 2) than websites that were not HONcode certified (mean 6.75, SD 2.7), this was not significantly different (*P*=.139). There was no significant difference in readability scores (*P*=.92) when comparing websites with HONcode seal status or lack thereof (mean 9.83, SD 1.9 and mean 9.91, SD 1.7, respectively).

## Discussion

This study found that overall, the quality of websites with statin-related information tended to be poor. The website content was not sufficiently comprehensive, and the reading level was too difficult for the average reader to fully comprehend. The credibility of the websites varied, although overall websites bearing the HONcode seal had higher credibility than those without.

Here, we formally assessed the quality of websites addressing statins and their side effects. The finding that the quality of information is of variable caliber is consistent with studies investigating web-based health information on other topics [4,17,75]. Although the criteria used by Google’s ranking algorithm is confidential, Google’s guidelines state that it uses a series of algorithms that account for the words of the query, relevance and usability of web pages, the expertise of sources, ease of use on mobile device interfaces, as well as location and settings to determine the results displayed [76,77]. However, this study demonstrates that the most prominent websites in the Google search ranking are not necessarily of high quality.

The lack of correlation between accuracy and credibility or readability is a concern if patients are using the information to understand their condition and take action related to it. Patients with poor health literacy may use inaccurate and untrustworthy information in deciding whether to see a health professional following the onset of symptoms or whether to undertake tests and treatments that may be unnecessary or recommended by health professionals [12]. Vulnerable populations are at higher risk of having poor health literacy and experiencing CVD [12,13], making them especially vulnerable to inaccurate, untrustworthy, and unreadable websites on statins.

While most websites analyzed as part of this study scored low in accuracy, this tended to be attributed to a lack of completeness of information rather than a lack of factual information. While the checklist developed here may be stringent, it would be reasonable to expect that websites dedicated to statins would be comprehensive. The lack of comprehensiveness in the information provided on the websites could result in consumers overlooking important details unless they browse through multiple websites. Furthermore, visits to multiple websites may not generate clarity but confusion. This is due to the increased likelihood of encountering inconsistent information, particularly as websites have different agendas based on the website type. That said, the commercial websites scored, on average, just as well as the not-for-profit websites, indicating that they can be a valuable source of information for consumers. It also indicates that government and other not-for-profit websites will be required to at least match the accuracy of commercial websites if they are to remain relevant in Google’s search algorithm, as having information-rich content is a factor that contributes to higher search rankings [78].

When browsing the internet, one would expect government and other not-for-profit websites to provide credible information. However, some of these websites returned relatively low credibility scores and overall were not significantly more credible than commercial websites. Over half of the 20 websites analyzed provided evidence-based information and avoided anecdotal evidence, increasing their credibility rating [16,44]; however, it was concerning that 5 websites provided information based on anecdotal evidence. As expected, media websites received low credibility scores as news articles about statins contained personal views and anecdotes. Other indicators of credibility were lacking by a large proportion of websites, in particular author qualifications and details about sponsorship, funding, and conflicts of interest [16,44]. Furthermore, many of the commercial and media websites included advertisements, including health-related advertisements [16], and 5 of them did not include the disclaimer that web-based health information does not replace a practitioner’s advice [44]. Thus, even patients with a degree of health literacy would find it difficult to accurately appraise the credibility of many of these websites on statins.

Given that the general public is unlikely to be fully equipped to gauge the credibility of web-based health information presented [12], clinicians could advise that patients identify the presence of the HONcode seal as this merits some confidence in the information presented [16]. However, the code does not necessarily imply that websites are comprehensive. Additionally, website developer application for the HONcode seal is voluntary, so high-quality websites may not bear the HONcode seal. The finding that the readability of the websites with the HONcode seal was not at a suitable level means that such websites may not represent digestible patient health information. Furthermore, a practical issue is that the HONcode seal is at the bottom of the web page and is thus not necessarily evident at first glance.

Many patient demographic groups have been found to read at a level more than 3 years below their completed educational years [79]. Thus, the study results may not be indicative of the severity of the problem posed by websites with high readability scores in terms of the general public’s understanding of web-based information [79]. Those with limited literacy skills tend to have poorer health status due to a lack of knowledge and understanding of health care issues and a diminished ability to participate in shared decision-making in the clinical context [80]. They also tend to have poorer compliance with treatment recommendations and subsequent disease progression, as well as a higher risk for seeking emergency care and more frequent and longer inpatient admissions [27].

Additionally, other factors besides readability play into the way a text is received, including logical and sequential presentation of information. Additionally, alternative media such as images and graphs provide a well-documented “picture superiority” effect that boosts understanding of and engagement with a text [81], although some of these may also require interpretation by consumers.

A limitation of this study of these websites is that the internet is dynamic, with websites updated at any time. The search used in undertaking the study is constrained temporally in its noniterative nature, as well as its method, which used only the major search engine Google. While a metasearch capturing results from multiple search engines would provide a more comprehensive view of the information about statins on the internet, it is unlikely to represent the behavior of the public [82]. Additionally, only 8 of the websites in the study were updated in some way after completion of this study, and the information on some websites is dated as more than 10 years old.

Overall, this study has demonstrated that within the surfeit of information available on the internet regarding statin therapy, the quality of websites is of mixed caliber. The content of information is generally accurate but incomplete, while credibility is variable. Readability is generally of a level too difficult for the general public to comprehend. This suggests a need for guidance to website developers of health care websites in order to capitalize on the vast potential of the internet to equip patients with the empowerment of improved health information and health literacy. It also highlights that clinicians will need to be educated themselves about what is on the internet and what constitutes accuracy, credibility, and readability in order to impart this knowledge to their patients. During the COVID-19 pandemic, the methods through which patients seek information about their health have shifted toward increasingly internet-based means, making the quality of information on the internet of particular significance in the current climate and for the foreseeable future.

## Appendix

Multimedia Appendix 1 Top 20 websites analyzed in the study.

## Abbreviations

CVD: cardiovascular disease
HONcode: Health on the Net Code of Conduct
SMOG: Simple Measure of Gobbledygook
