# Supplementary material for: Evaluation of the Accuracy, Credibility, and Readability of Statin-Related Websites: Cross-Sectional Study
Source: Interact J Med Res. 2024 Mar 14;13:e42849. doi: 10.2196/42849 (PMC10979333; doi:10.2196/42849)
Supplement: Multimedia Appendix 1 [file ijmr_v13i1e42849_app1.docx]

| Search rank | Website | Website type | HONcode certified |
| --- | --- | --- | --- |
| 1 | MedicineNet.com [55] | Commercial | Yes |
| 2 | Healthdirect.com [56] | Not-for-profit | Yes |
| 3 | Wikipedia.com [57] | Not-for-profit | No |
| 4 | WebMD.com [58] | Commercial | Yes |
| 5 | Health.com [59] | Commercial | No |
| 6 | MedlinePlus.gov [60] | Not-for-profit | No |
| 7 | Healthengine.com [61] | Commercial | No |
| 8 | NHS.uk [62] | Not-for-profit | No |
| 9 | Mayo Clinic.org [63] | Not-for-profit | No |
| 10 | Healthline.com [64] | Commercial | Yes |
| 11 | Medicalnewstoday.com [65] | Commercial | Yes |
| 12 | Mercola.com [66] | Commercial | No |
| 13 | NHS.uk [67] | Not-for-profit | No |
| 14 | Statinadverseeffects.com [68] | Not-for-profit | No |
| 15 | HeartUK.org [69] | Not-for-profit | No |
| 16 | Telegraph.co [70] | Media | No |
| 17 | NPS.org [71] | Not-for-profit | Yes |
| 18 | Rxlist.com [72] | Commercial | Yes |
| 19 | Drugs.com [73] | Commercial | Yes |
| 20 | CNN.com [74] | Media | No |
